# Magnetic resonance imaging assessed enteric motility and luminal content analysis in patients with severe bloating and visible distension

**DOI:** 10.1111/nmo.14381

**Published:** 2022-04-19

**Authors:** Ruaridh M. Gollifer, Stuart A. Taylor, Alex Menys, Natalia Zarate‐Lopez, Dave Chatoor, Anton Emmanuel, David Atkinson

**Affiliations:** ^1^ Centre for Medical Imaging University College London (UCL) London UK; ^2^ Department of Gastroenterology University College London Hospitals London UK

**Keywords:** bloating, dynamic MRI, dysmotility, IBS‐C, texture analysis

## Abstract

**Background:**

Gastrointestinal symptoms in functional gut disorders occur without any discernible structural gut abnormality. Preliminary observations on enteric MRI suggest possible abnormal content and motility of the terminal ileum (TI) in constipation‐predominant IBS (IBS‐C) with severe bloating, and in functional bloating and distension (FABD) patients. We investigated whether MRI can quantify differences in small bowel (SB) content and motility between patients and healthy controls (HCs).

**Methods:**

11 IBS‐C (mean age 40 [21–52] years; 10 women) and 7 FABD (36 [21–56]; all women) patients with bloating and 20 HCs (28 [22–48]; 6 women) underwent enteric MRI, including dynamic motility and anatomical sequences. Three texture analysis (TA) parameters assessed the homogeneity of the luminal content, with ratios calculated between the TI and (1) the SB and (2) the ascending colon. Four TI motility metrics were derived. Ascending colon diameter (ACD) was measured. A comparison between HCs and patients was performed independently for: (1) three TA parameters, (2) four TI motility metrics, and (3) ACD.

**Key Results:**

Compared with HCs, patients had TI:colon ratios higher for TA contrast (*p* < 0.001), decreased TI motility (lower mean motility [*p* = 0.04], spatial motility variation [*p* = 0.03], and area of motile TI [*p* = 0.03]), and increased ACD (*p* = 0.001).

**Conclusions and Inferences:**

IBS‐C and FABD patients show reduced TI motility and differences in luminal content compared with HCs. This potentially indicates reflux of colonic contents or delayed clearance of the TI, which alongside increased ACD may contribute to symptoms of constipation and bloating.

## Graphical abstract

Enteric MRI identifies discrete changes in enteric content and motor physiology which may contribute to the symptom of severe bloating in IBS‐C and functional bloating patients. Enteric MRI offers a potential biomarker of response to therapeutic interventions.

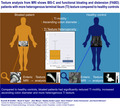

**Key Points**

- A heterogeneous feces‐like appearance (“fecalization”) was seen on MRI in the terminal ileum luminal content of IBS‐C and FABD patients.
- Terminal ileum MRI motility was lower, less varied, and less active in IBS‐C and FABD patients.
- Increased right colonic diameter seen on MRI in IBS‐C and FABD patients may also contribute to abdominal bloating.

## 1 INTRODUCTION

Bloating and distension have been reported by 30% of the general population and up to 95% of patients with functional gastrointestinal disorders.^1^ Bloating refers to the subjective sensation of increased abdominal pressure and fullness, particularly during the postprandial period, and distension refers to an objective increase in abdominal girth.^1^, ^2^, ^3^, ^4^ Not all patients with bloating have distension.

Irritable bowel syndrome (IBS) is a condition characterized by chronic gastrointestinal symptoms including abdominal pain, bloating, and altered bowel habit.^5^, ^6^ The etiology of IBS is multifactorial with altered gut motility, visceral hypersensitivity, and dysfunction of the brain–gut axis having been suggested.^5^ There is no recognized biomarker, and patients usually have structurally normal bowel on standard investigations such as cross‐sectional imaging and endoscopy.^7^ Approximately 44% of IBS patients with a sensation of bloating also describe abdominal distension.^8^ Bloating with visible distension is more commonly seen in patients with IBS‐C than with IBS‐D.^1^ Furthermore, distension is frequently seen in patients with associated constipation and slow colonic transit.^9^

Rome IV defines functional bloating and/or distension (FABD) as recurring bloating and distension occurring on average >1 day per week, with bloating and distension as the predominant symptoms in individuals not meeting diagnostic criteria for other functional gut disorders.^6^ Thus, there are patients with abdominal bloating and distension who have normal bowel habit and do not fit the IBS pattern.

Whatever the underlying Rome IV patient classification, bloating is a pervasive symptom extremely difficult to treat and negatively contributing to patients’ quality of life.^10^

There are various theories about the cause of bloating, including increased colonic fecal content, excessive diaphragmatic descent, or altered perception of abdominal sensations by the patient.^10^, ^11^, ^12^ Abnormal transit, constipation, and increased colonic diameter are also postulated to contribute to symptoms,^13^, ^14^, ^15^, ^16^ although remain controversial.

**FIGURE 1:**
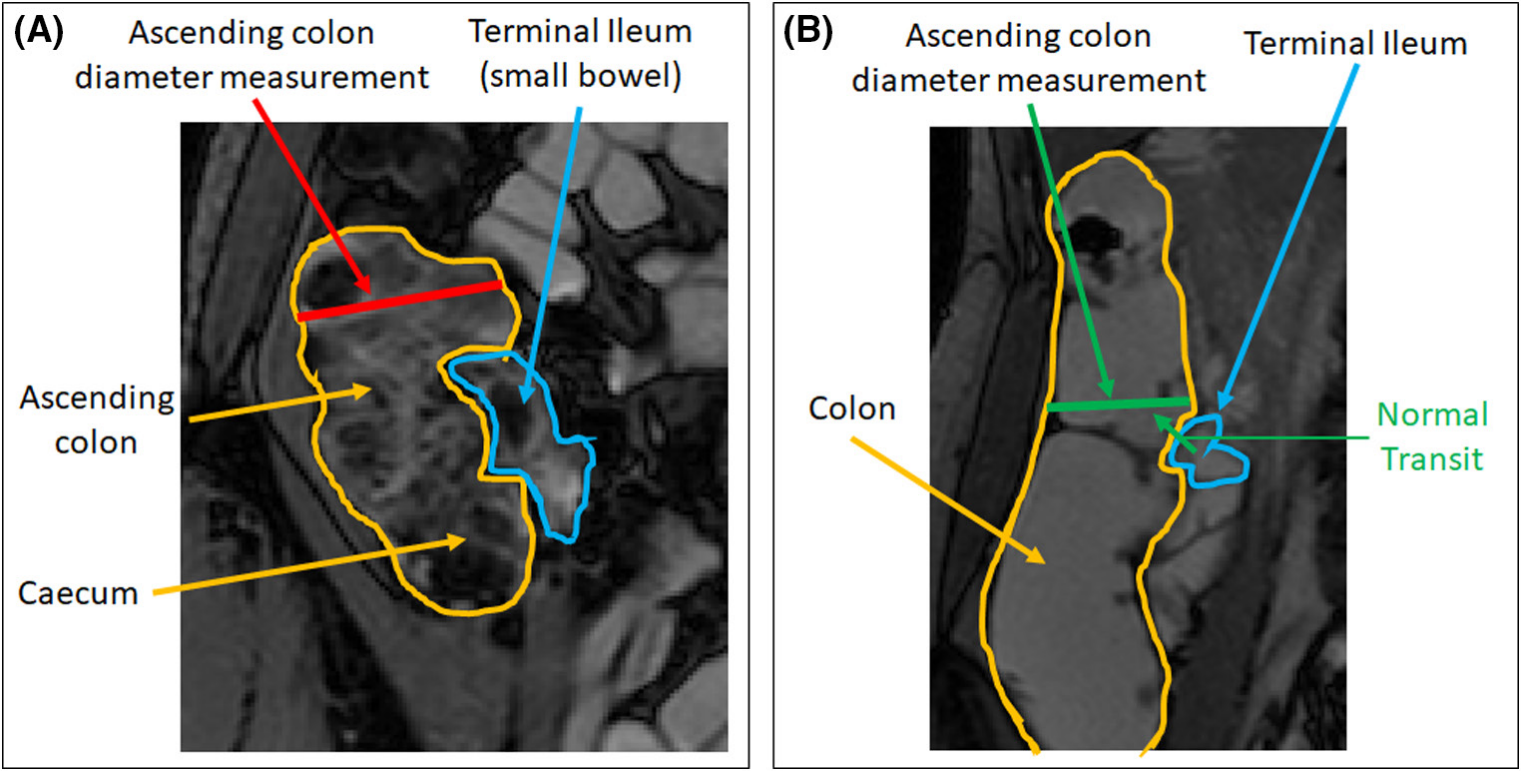
Fecalization of the terminal ileum with ascending colon diameter measurement (red) in a patient presenting with distension (A) vs normal terminal ileum with ascending colon diameter measurement (green) in healthy control where the normal transit (in green) would be from the terminal ileum (blue) into ascending colon and cecum (orange) (B)

Enteric MRI is increasingly used in patients with functional gut disorders, not least to exclude structural causes for symptoms. In the authors’ practice, a pattern of “fecalization” of terminal ileum luminal content in patients with IBS‐C and functional bloating has been anecdotally reported, that is, a feces‐like appearance within the terminal ileum instead of the expected homogeneous oral contrast agent, possibly due to delayed ileal clearing or reflux of cecal contents (Figure 1).

It is now possible to capture bowel motility patterns and quantify content noninvasively using enteric MRI,^13^, ^17^, ^18^, ^19^, ^20^, ^21^, ^22^, ^23^, ^24^, ^25^, ^26^, ^27^, ^28^, ^29^, ^30^, ^31^, ^32^ although differences in enteric motility and terminal ileum luminal content have not previously been investigated in patients with functional bowel disorders. MRI has potential to aid in phenotyping these patients more accurately and increase our understanding of its pathophysiology, and influence clinical management. In addition, measuring colonic caliber is not routinely done in all patients and there are little data on the colonic diameter in a cohort of patients with distension.

Based on these clinical observations, the purpose of this retrospective study was to investigate whether MRI can demonstrate differences in terminal ileum luminal content, motility, and regional colon diameter between patients presenting with severe abdominal bloating and visible distension of functional etiology, and healthy controls.

## 2 MATERIALS AND METHODS

### 2.1 Patient selection

The current study was approved on October 18, 2019, by the London‐Hampstead Research Ethics Committee (IRAS ID: 248064, REC Reference 19/LO/1586).

#### 2.1.1 Study overview

Patient data were collected retrospectively at the University College London Hospital (UCLH) from MRI scans performed as part of usual clinical care between December 2012 and June 2018. The MRI was performed on clinical grounds for patients where there was concern about intra‐abdominal pathology and to exclude structural conditions such as pseudo‐obstruction, given the presenting symptom of severe distension and bloating. The requirement for consent was waived for the retrospective analysis in this study.

Healthy control data from volunteers were included from a prior study designed to assess the repeatability in human volunteers of software‐quantified MRI small bowel motility.^32^ The healthy control subjects provided written informed consent for the original research study, and the requirement for consent was waived for the retrospective analysis in this study.

#### 2.1.2 Recruitment criteria

Patients specifically presenting with severe objective abdominal distension and bloating were identified from a specialist neurogastroenterology clinic for study inclusion. All patients had undergone enteric MRI with no organic or structural cause of the abdominal distension identified. Distension was unresponsive to routine clinical management, including the low FODMAP diet and standard and advanced laxative regime for those patients with altered bowel habit. All patients had normal celiac antibody tests and a negative glucose breath test.

The final diagnosis for each patient was based on the Rome IV classification of lower GI functional gut disorders.^5^, ^6^

Healthy controls (*n* = 20) were nonsmokers and abstained from caffeinated and alcoholic drinks on the day of the enteric MRI and any motility influencing medication for at least 1 week before. Exclusion criteria were any known chronic intestinal disease, self‐reported gastrointestinal (GI) symptoms, history of GI surgery or use of any long‐term medication excluding the oral contraceptive.

### 2.2 MRI protocol

Subjects fasted for 4 h before slowly ingesting 1 L of 2% mannitol solution, starting from 40 to 50 min prior to the start of the scan to distend the small bowel.

Both healthy controls and bloated patients were scanned in the prone position. This is part of the center's standard enteric MRI protocol to try to improve bowel distension and reduce breathing artifacts.

In both patients and controls, a dynamic “cine motility” sequence was acquired during a 20‐second breath‐hold with a temporal resolution of 1 image (or volume) per second prior to the administration of the antispasmodic butylscopolamine for subsequent anatomical sequence acquisition (Table 1). Coronal blocks were repeated to encompass the whole small bowel volume (Table 1).

**TABLE 1:** Balanced sequence parameters for motility and anatomical MRI data. All subjects were scanned in prone position. Motility was captured using a 2D balanced turbo field‐echo sequence in healthy controls and a 2D coronal, balanced steady‐state free precession sequence in patients

MR parameter	Motility		
	HC (Philips Achieva)	Patients (Philips Achieva)	Patients (Siemens Avanto)
Scan type	Dynamic	Dynamic	Dynamic
Field strength	3T	3T	1.5T
TR (ms)	3.5	3.7	3.6–4.3
TE (ms)	1.7	1.8	1.8–2.2
Flip angle	20	20	47–64
Field of view (mm)	420 × 420	Variable	Variable
Reconstructed spatial resolution (mm)	2.5 × 2.5	2.5 × 2.5	2.5 × 2.5
Slice thickness (mm)	10	5	10

MR parameter	Anatomical		
	HC (Philips Achieva)	Patients (Philips Achieva)	Patients (Siemens Avanto)
Scan type	BTFE	BTFE	FISP
Field strength	3T	3T	1.5T
TR (ms)	2.5	2.5	3.5–4.3
TE (ms)	1.2	1.2–1.3	1.5–1.9
Flip angle	45	45	46
Field of view (mm)	400 × 400	268 × 224	256 × 166
Acquired spatial resolution (mm)	1.5 × 2	1.5 × 2	1.5 × 2
Reconstructed spatial resolution (mm)	1 × 1	1 × 1	1 × 1
Slice thickness (mm)	5	5	4

Abbreviations: BTFE, balanced turbo field echo; FISP, fast imaging with steady‐state precession; HC, healthy control; MRI, magnetic resonance imaging; TE, echo time; TR, repetition time.

There were three parts to the study: texture analysis to quantify the properties of luminal content; terminal ileal motility analysis; and ascending colon diameter comparison, all performed blinded to the patient/control group. The primary endpoint was the texture analysis comparison between patients and healthy controls.

All analysis was performed in MATLAB 2018 (The MathWorks).

### 2.3 Texture analysis

Texture analysis (TA) quantifies the variation in image intensities or gray levels within a region of interest (ROI) by calculating Haralick's gray‐level co‐occurrence matrix (GLCM).^33^ A full description of TA is given in Figure S1.

**FIGURE 2:**
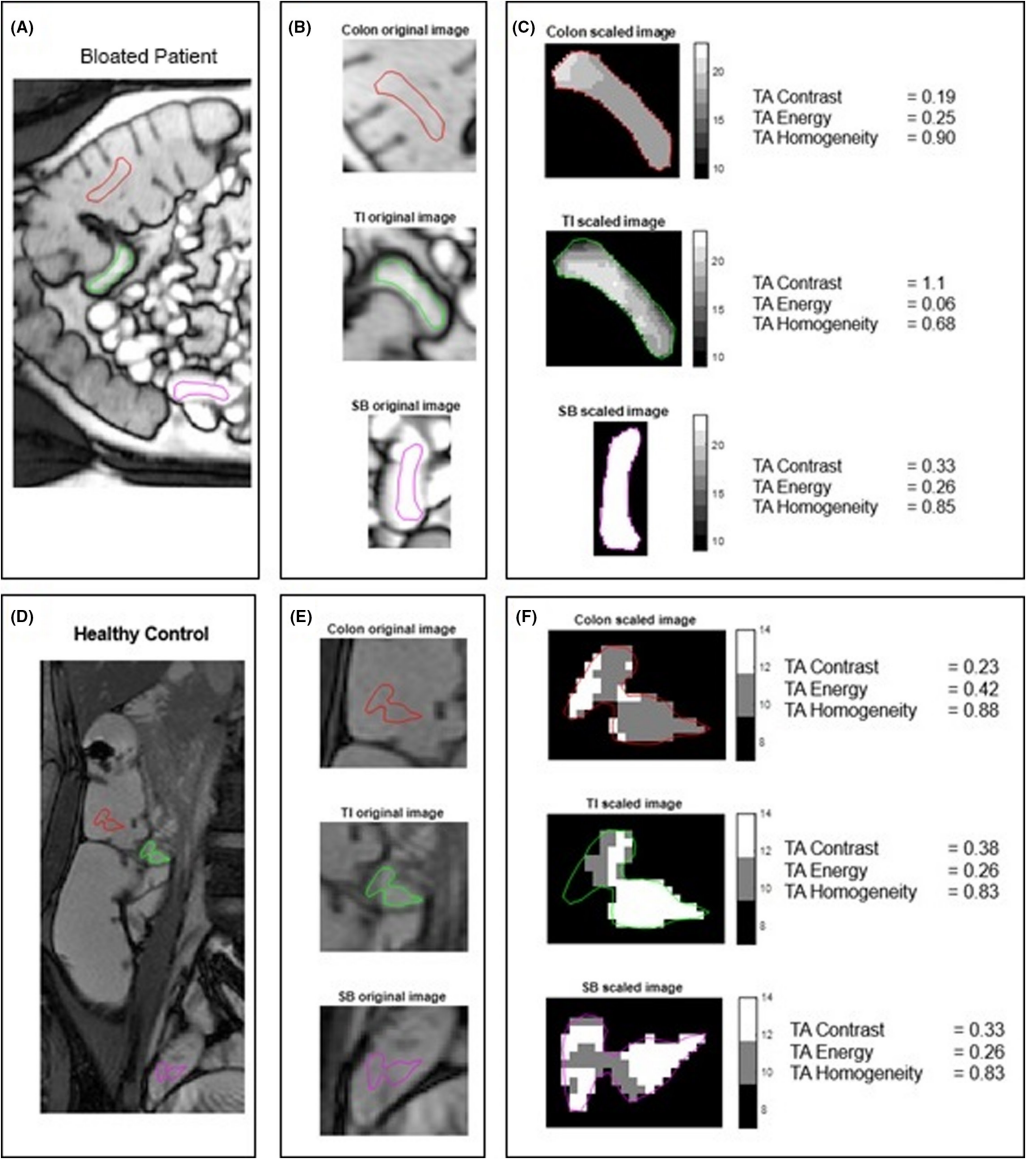
ROI is drawn on the original MRI image (A–B for a patient with distension and D–E for a healthy control), and this ROI is scaled between 0 and 32 gray levels (C and F). Shown here is a representative patient with distension, with the TI that is patchy/heterogeneous with a large variation in gray‐level values as indicated by a TA contrast of 1.1 and the small bowel and colon, which are more homogeneous with fewer gray‐level values as indicated by a TA contrast of 0.33 and 0.19, respectively (C). A representative HC with homogeneous luminal contents throughout the SB and into the colon is shown with a low number of gray levels in the TI, SB, and colon, indicated by similar TA contrasts of 0.38, 0.33, and 0.23 (F)

A study coordinator (research fellow) with 4 years of training in enteric MRI (RG) placed three ROIs of identical shape and size on anatomical BTFE (balanced turbo field echo) and FISP (fast imaging with steady‐state precession) images: (1) in the TI (in a single slice where the TI was most visible), (2) in a proximal region of small bowel (bright on T2‐weighted images and well distended, i.e., filled with oral contrast and not collapsed), and (3) in the ascending colon (5–10 cm above the ileocecal valve and in the center of the colon, i.e., close to the TI ROI, but at a standard distance away in all controls and patients) (Figure 2).

Because texture analysis measures are affected by the shape and size of the region being analyzed, the ROI shape was determined by drawing the ROI in the smallest of the three regions and then copying it to the other two locations.

Haralick's gray‐level co‐occurrence matrices (GLCMs) were calculated in four directions (0°, 45°, 90°, and 135°) for pixel distances from 1 to 4 (distance between the pixel of interest and its neighbor) using 32 gray levels.

Three GLCM summary measures (TA contrast, TA energy, and TA homogeneity^33^) were derived to quantify the heterogeneity in the ROIs (averaged over the four directions).

For each summary measure, the ratio was calculated between the TI and (1) a proximal part of the SB and (2) the ascending colon for each of the 4‐pixel distances (Figure 2).

The texture contrast is a measure of local image variations, that is, a measure of the intensity contrast between a pixel and its neighbor with a contrast of 0 for a constant image (contrast range: 0 to [number of gray levels‐1]^2^).

Texture analysis energy (TA energy) provides the sum of squared elements in the GLCM and has a range of values from 0 to 1 (for a constant image).

Texture analysis homogeneity (TA homogeneity) has a range of values from 0 to 1 and gives a measure of the closeness of the distribution of the elements in the GLCM to the GLCM diagonal.

Regions of interest with a heterogeneous texture, indicating a mixture of different luminal contents, will have a high TA contrast, a low TA energy (nearer to 0), and a low TA homogeneity (nearer to 0), and vice versa for a ROI in more homogeneous bowel contents.

The TI/SB and TI/colon ratios will indicate whether the texture in the TI compared with the SB or the colon is (i) equally homogeneous or equally heterogeneous (TA contrast ratio = 1), (ii) more heterogeneous (TA contrast ratio >1), or (iii) more homogeneous (TA contrast ratio <1).

### 2.4 Terminal ileum motility assessment

For each 2D cine motility sequence, motility was quantified using a previously validated optic flow‐based registration technique^17^ (GIQuant, Motilent).

The motility quantification automatically selects a reference time frame for each slice. The study coordinator (RG) selected the slice where the terminal ileum was most visible with the reference frame from that slice used for ROI placement. The ROIs were validated by a research fellow with 8 years of enteric MRI experience (AM).

Subjects were excluded from the motility analysis if data were unavailable, or the terminal ileum was not able to be identified on any of the available slices.

The motility metrics were based on a previous study investigating motility in Crohn's disease^21^ and in this study were derived from the single TI ROI (See Figure S2, which describes the motility metrics):

1. mean motility
2. spatial variation of motility
3. temporal variation of motility
4. area of motile TI

### 2.5 Ascending colon diameter assessment

The colon diameter was measured by the study coordinator (RG) from a position on the ascending colon wall 5–10 cm vertically above the ileocecal valve to the opposite ascending colon wall and along a line perpendicular to the long, vertical axis of the ascending colon (Figure 1).

### 2.6 Statistical analysis

All data were checked for normality using a Shapiro–Wilk test (alpha = 0.05).

All exclusions were confirmed prior to the final data analysis.

If both the healthy control group and patient group data were normally distributed, an independent two‐sample *t* test was performed for comparison. If one of the groups was not normally distributed, the Mann–Whitney test was performed.

Either a two‐sample *t* test (for normal data) or the Mann–Whitney test (for non‐normal data) was performed to compare each metric between healthy controls and patients, with *p* < 0.05 indicating significance. For the TA summary measures, the Bonferroni correction was used to account for the multiple comparisons at the four‐pixel distances for each measure, with *p* < 0.0125 (*p* < 0.05/4) being taken as statistically significant.^34^

Additionally, either a two‐sample *t* test (for normal data) or the Mann–Whitney test (for non‐normal data) was performed to compare IBS‐C patients, FABD patients, and HCs for the TI/small bowel and TI/colon TA contrast measure for a pixel distance of 1.

## 3 RESULTS

### 3.1 Cohort demographics

**FIGURE 3:**
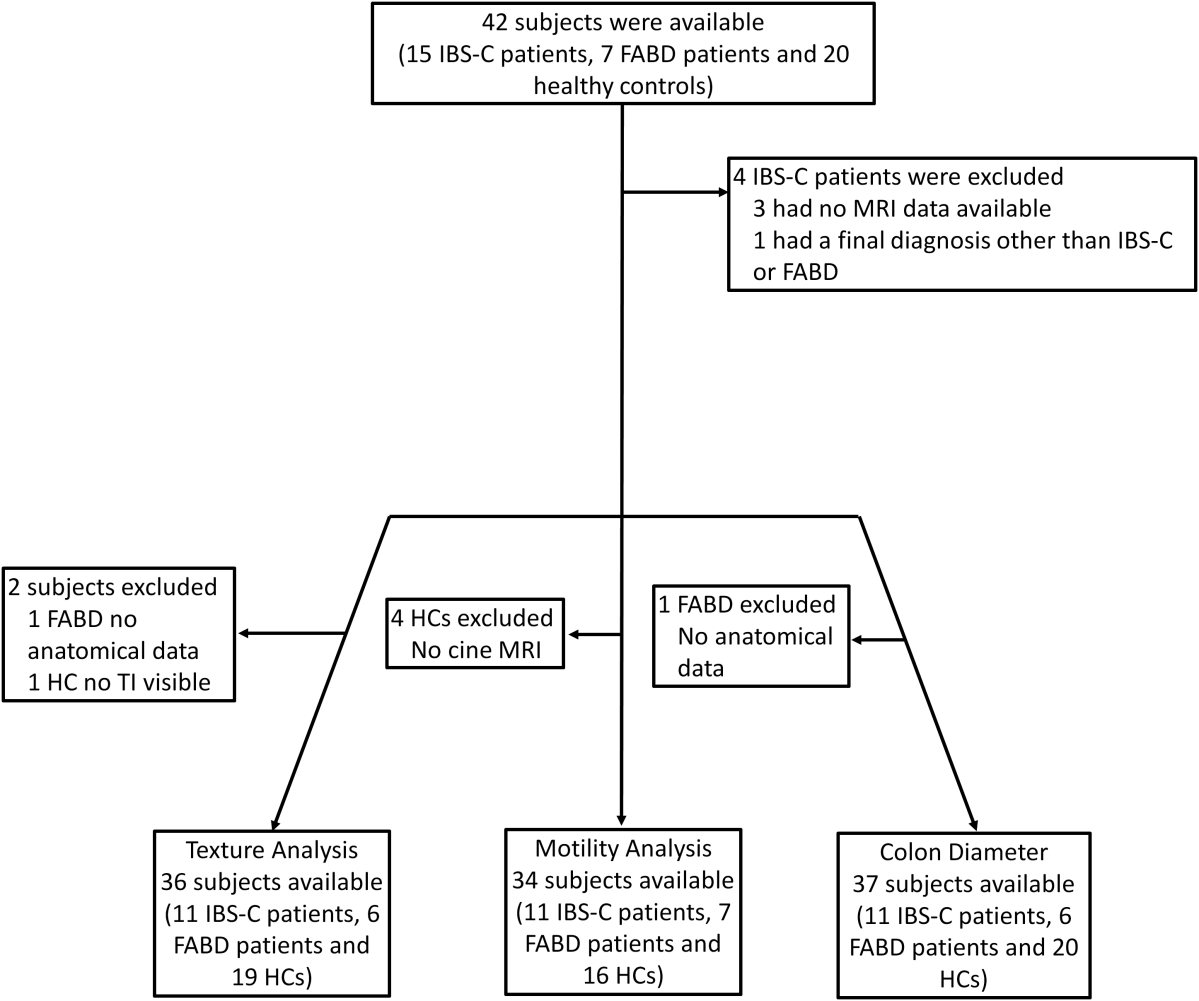
Flowchart demonstrating patient inclusions and exclusions for texture analysis, motility analysis, and colon diameter comparison

The full study cohort consisted of 42 subjects: fifteen patients fulfilling Rome IV criteria for IBS‐C, 7 patients fulfilling Rome IV criteria for functional abdominal bloating and/or distension (FABD), and 20 healthy controls (Figure 3).

Four IBS‐C patients were excluded due to insufficient MRI data (*n* = 3) and a subsequent diagnosis of chronic intestinal pseudo‐obstruction (CIPO) (*n* = 1), leaving 11 IBS‐C (mean age, 40; age range, 21–52 years; 10 women) and 7 functional bloating patients (mean age, 36; age range, 21–56 years; all women) and 20 healthy controls (mean age, 28; age range, 22–48 years; 6 women) available for analysis. Ten patients (all with IBS‐C) had undergone bowel retraining as part of their bowel dysfunction management. Severe symptoms persisted in spite of this intervention. No patients with functional abdominal bloating and distension were referred for bowel retraining. A colonic transit study was performed only on 3 patients with IBS‐C.

Additionally, 2 subjects were excluded from TA, 4 subjects from the motility analysis, and 1 subject from the ascending colon diameter analysis as per protocol stipulations described above (Figure 3).

Failure of oral contrast to reach the TI was observed in 9 of 20 HCs and 4 of 18 patients.

### 3.2 Texture analysis

#### 3.2.1 TI/SB ratio comparison of patients vs healthy controls using texture analysis measures

##### TA contrast

The best TI‐to‐SB ratio (TI/SB ratio) TA parameter to discriminate between the patients and healthy controls was TA contrast at a pixel distance of 1 pixel (or 1 mm) (See Table S1, which provides the raw TA data). The TI/SB ratio was higher in patients (mean TI/SB ratio of 2.28, *n* = 17) than in HCs (mean TI/SB ratio of 1.56, *n* = 19), but this did not reach statistical significance (*p* = 0.08) (See Figure S3, which shows the TA contrast TI/SB ratio).

##### TA energy and homogeneity

There were also no significant differences between patients and HCs for the TI/SB ratio (see Table S1, which provides the raw TA data) for TA energy (see Figure S4, which shows the TA energy TI/SB ratio) or TA homogeneity measures (see Figure S5, which shows the TA homogeneity TI/SB ratio).

#### 3.2.2 TI/colon ratio comparison of patients vs healthy controls using texture analysis measures

##### TA contrast

**FIGURE 4:**
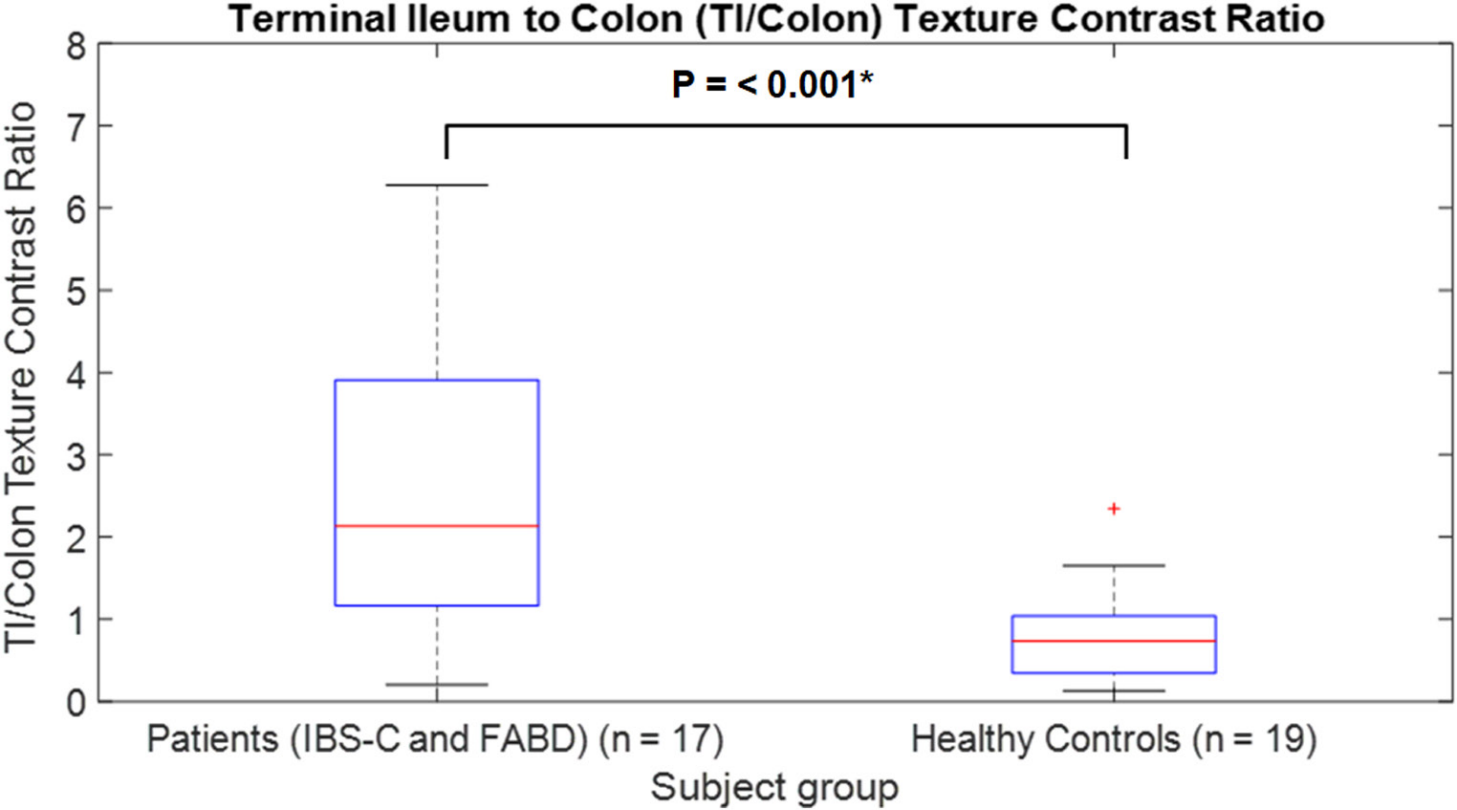
Boxplot of terminal ileum‐to‐colon (TI/colon) texture contrast ratio at a pixel distance of 1 pixel for patients vs healthy controls. *indicates a significant result

The best TA measure to discriminate between the patients and HCs for the TI‐to‐colon ratio (TI/colon ratio) was TA contrast at a pixel distance of 1 pixel (*p* < 0.001) (see Table S1, which provides the raw TA data). The TI/colon ratio was significantly higher in patients (mean TI/colon ratio of 2.8, *n* = 17) than in HCs (mean TI/colon ratio of 0.82, *n* = 19) (Figure 4).

##### TA energy

There was a significant difference between patients and HCs for the TI/colon ratio for TA energy at a pixel distance of 1 (*p* = 0.01) (See Figure S6, which shows the TA energy TI/colon ratio). The TI/colon ratio was significantly lower in patients (mean TI/colon ratio = 1.0) than in HCs (mean TI/colon ratio = 2.7).

##### TA homogeneity

There were significant differences between patients and HCs for the TI/colon ratio for TA homogeneity at a pixel distance of 1 (*p* < 0.001) (See Figure S7, which shows the TA homogeneity TI/colon ratio). The TI/colon ratio was significantly lower in patients (mean TI/colon ratio = 0.92) than in HCs (mean TI/colon ratio = 1.1).

#### 3.2.3 IBS‐C, FABD, and HC comparisons using texture analysis contrast measure

The findings for TI/SB TA contrast ratio or the TI/colon TA contrast ratio at a pixel distance of 1 for FABD (*n* = 6) vs HCs (*n* = 19) follow that of the whole patient cohort (IBS‐C and FABD) (*n* = 17) vs HCs. No significant differences were found between IBS‐C (*n* = 11) and FABD patients. There was no significant difference between IBS‐C and HCs (*p* = 0.016) (Figure S8).

### 3.3 Terminal ileum (TI) motility

A summary of automated motility metrics for the patients and healthy controls is shown in Table 2.

**TABLE 2:** Median, minimum, and maximum automated motility metric values for patients and healthy controls. *p*‐values for the differences between patients and HCs (patients had lower values for all motility metrics)

Motility metrics	Patients (*n* = 18)			Healthy controls (*n* = 16)			Patients vs. HCs
	Median	Range		Median	Range		*p*‐values
		Min.	Max.		Min.	Max.	
Mean motility	0.230	0.122	0.433	0.311	0.136	0.629	0.04*
Spatial variation	0.072	0.006	0.198	0.107	0.045	0.350	0.03*
Temporal variation	0.007	0.001	0.021	0.018	0.003	0.061	0.06
Area of motile TI (% of ROI)	92.4	48.9	100.0	98.7	46.3	100.0	0.04*

Abbreviations: HC, healthy control; IBS‐C, irritable bowel syndrome with constipation; Max., maximum; Min., minimum.

* Indicates a significant result.

**FIGURE 5:**
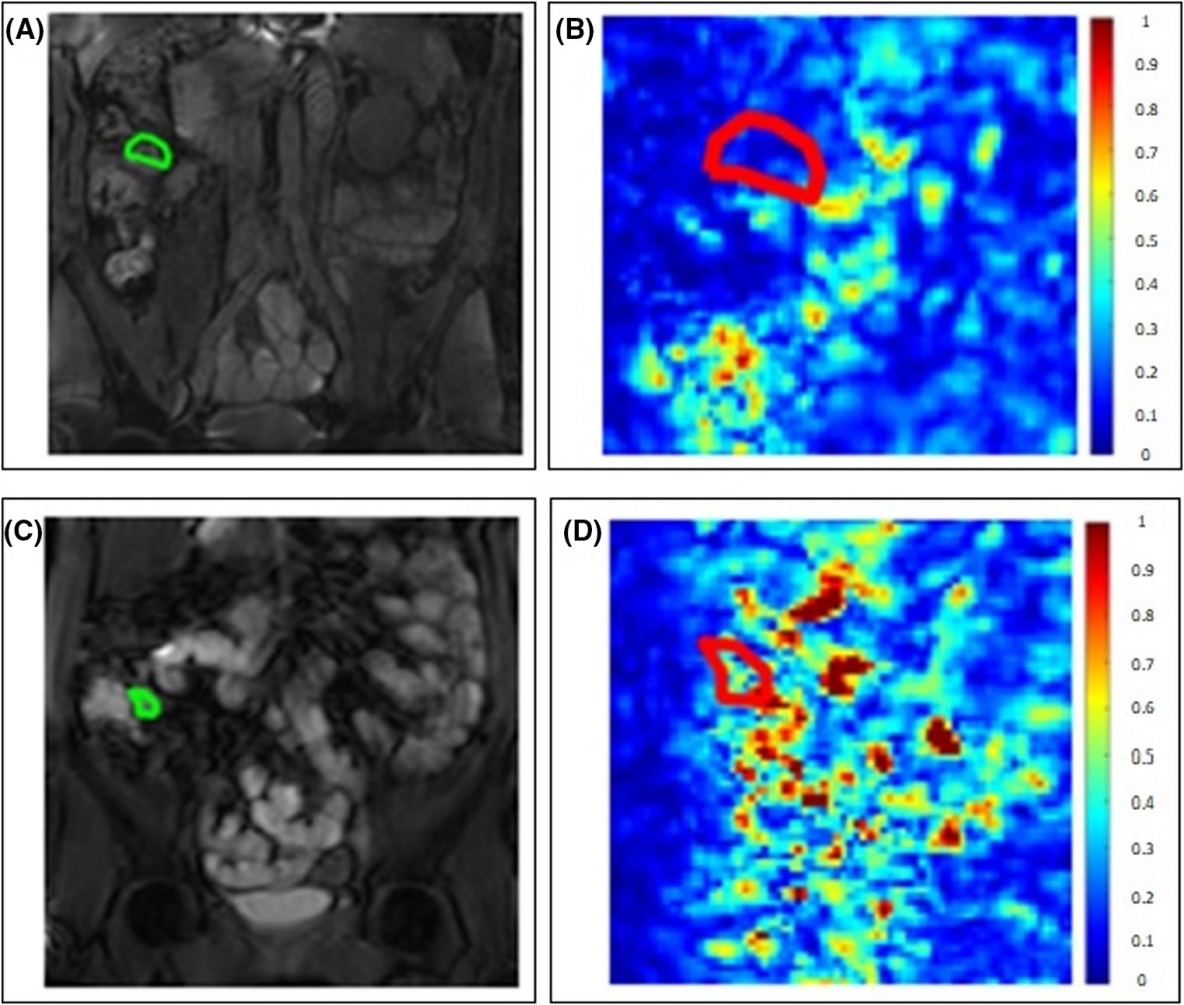
Two examples of coronal motility maps based on the standard deviation of the Jacobian determinant with color bar showing low (blue) to high (red) motility. A region of interest was drawn in the terminal ileum on the reference frame (A and C) with low motility seen in the patient with distension (B) and high motility seen in the healthy control (D)

There were significant differences between patients (*n* = 18) and HCs (*n* = 16) with lower TI mean motility (metric 1, median of 0.23 in patients compared with 0.31 in HCs, *p* = 0.04), TI spatial variation of motility (metric 2, median of 0.07 in patients compared with 0.11 in HCs, *p* = 0.03), and area of motile TI (metric 4, median of 92.4% in patients compared with 98.7% in HCs, *p* = 0.04) (Figure 5).

TI temporal variation (metric 3) was also lower in patients, compared with HCs, but this result was not significant (median of 0.007 in patients compared with 0.018 in HCs, *p* = 0.06).

### 3.4 Ascending colon diameter

**FIGURE 6:**
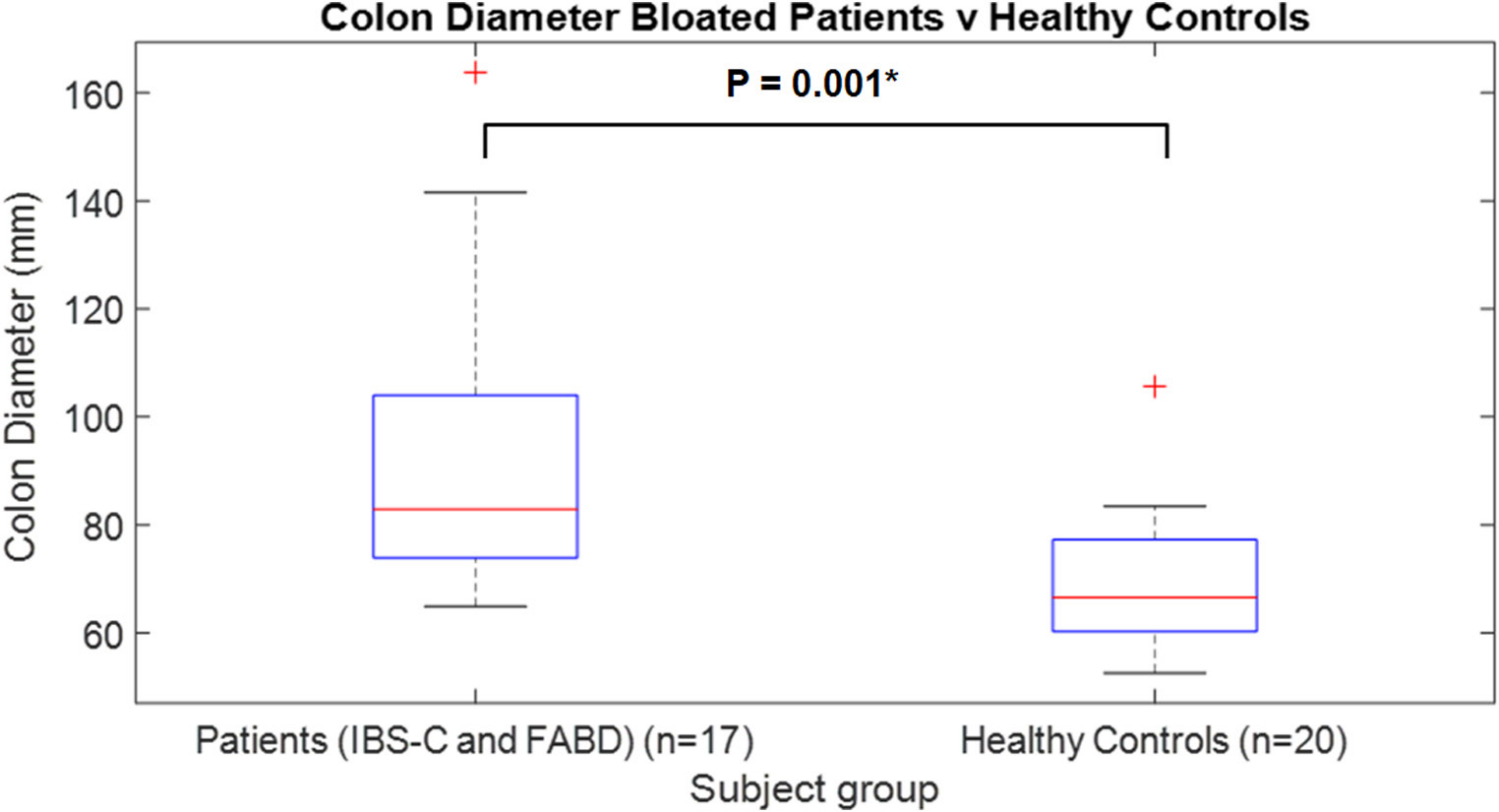
Boxplot of ascending colon diameter for patients vs healthy controls. *indicates a significant result

There was a significantly increased ascending colon diameter (*p* = 0.001) for the patient group (mean diameter = 92.6 mm) compared with healthy controls (mean diameter = 69.7 mm) (Figure 6).

## 4 DISCUSSION

This exploratory study suggests that MRI can identify differences in enteric content, motility, and colonic diameter between patients with functional abdominal bloating and distension and healthy controls. Specifically, we found differences in the TI‐to‐colon ratio of texture analysis summary measures, lower mean terminal ileal motility, spatial variability of motility and area of motile TI, and an increased ascending colon diameter in patients.

A TI/SB or TI/colon TA contrast ratio over 1 would indicate “fecalization” in the terminal ileum, assuming a homogeneous SB or colon. “Fecalization” describes the appearance of semi‐solid content in the TI caused by a mixture of liquid (brighter image intensity from chyme and/or mannitol contrast solution) and solid content (darker image intensity from solid/gas contents, i.e., feces‐like). These features would not be visible on a plain abdominal X‐ray. In most HCs, the contents within the small bowel, including the terminal ileum, and the cecum and first part of the ascending colon had a bright, homogeneous appearance with a smooth texture due to oral contrast filling and lack of solid contents. In other HCs, where the oral contrast did not reach the TI, there was still a homogeneous appearance in the TI, albeit with slightly less bright content likely due to chyme passage due to the proximal oral contrast load. In these cases, the colon either followed a similarly homogeneous pattern or was more heterogeneous in texture. Conversely, in our group of patients with IBS‐C and FABD the texture within the terminal ileum often appeared heterogeneous. Therefore, the higher the TA contrast ratio, the higher the degree of “fecalization” is likely in the terminal ileum. It should be noted that there may be some cases where there is “fecalization” in both the terminal ileum and the colon ROI, and therefore, the ratio would be around 1 and the terminal ileum “fecalization” would be missed (see Table S2).

We found that, out of our primary endpoints, the TI/colon ratio was better than the TI/SB ratio to discriminate between patients and HCs. This could be due to a more consistent anatomical location of the colon ROIs compared with the variable placement of the SB ROIs. Additionally, a better and more homogeneous content (a lack of solid/gas content due to the presence of either liquid chyme or oral contrast) in the TI compared with either an equally homogeneous or more heterogeneous content in the ascending colon in HCs, due to normal transit, could also in part explain the differences between patients and HCs. Due to the difficulty of the SB ROI placement without including bowel wall, it was not possible to always place the ROIs in the same part of the small bowel, that is, the ileum or the jejunum across all patients, so therefore there could be texture differences between different parts of the small bowel before the TI (see Figure S9).

The heterogeneous texture often observed within the terminal ileum of patients with IBS‐C and FABD could potentially be due to the reflux of cecal contents back into the terminal ileum, suggestive of slower progression of colonic contents in the aboral direction. In the current study, there were a few anecdotal examples where potential reflux of content could be seen on the motility scan of patients. However, due to the short motility scan times, it was difficult to determine whether this effect was occurring consistently.

Another explanation for differing TI content texture in patients with IBS‐C and FABD, compared with HCs, could be inhibition of TI emptying by fecal loading of the right colon leading to stasis of content, subsequent dehydration, and the “fecalization” appearance.

Texture analysis comparisons (particularly TI/SB contrast ratio and TI/colon contrast ratio at a pixel distance of 1) between the FABD patients and the HCs show a similar pattern to the findings between the whole patient cohort (FABD and IBS‐C) and HCs. There was no significant difference found between just IBS‐C patients and HCs suggesting unexpectedly that “fecalization” is more apparent in the FABD cohort than in the IBS‐C cohort.

The lower mean TI motility and area of motile TI found in patients supports the concept of ineffective transit of content from the small bowel into the colon. Indeed, this and the lower spatial variability of motility in patients may in part explain patient symptoms (particularly bloating and constipation). It has been repeatedly demonstrated that healthy small bowel shows heterogeneous motility on MRI and loss of this heterogeneity is abnormal.^20^, ^21^, ^35^

Disturbed gastrointestinal motility has been considered to play a role in IBS, but “signature” motility patterns have been hard to define. Delayed transit in association with IBS‐C has been reported,^36^, ^37^ and IBS‐C patients with delayed transit show greater abdominal distension than those with normal transit.^36^ Altered motility, in addition to increased total and segmental colonic transit time, has been shown to be linked to IBS symptoms.^38^, ^39^

Furthermore, postprandial motor activity is shorter in IBS patients than in HCs and migrating motor complex intervals are longer; that is, they occur less frequently in IBS‐C than in IBS‐D.^40^ This study provides a further example of TI motility as a potential biomarker in a functional GI‐related disease. No underlying gut motility disorder has been identified in IBS‐C patients; however, altered intestinal gas transit has been found previously in patients presenting with functional bloating and distension.^9^ It is possible that altered motility and TI filling may cause aberrant viscero‐somatic reflexes, resulting in abdominal distension and the sensation of bloating or fullness.^41^

Drugs that accelerate transit and reduce the volume of fecal matter within the gut lumen might improve symptoms in patients with IBS‐C with associated bloating. Clinical improvement was not observed in patients with IBS‐C referred for bowel retraining. The pharmacological approach to patients with functional bloating and otherwise normal or not constipated bowel habit has not been established. Studying the variation in symptomatic response to such drugs may help identify motility phenotypes to help target future pharmacological development.

While we did not measure global small bowel or colonic transit time in the current study, the progress of an oral contrast load through the small bowel does seem to give an indication of aberrant enteric function in the TI. It is possible the observed low TI motility reflects the failure of the oral contrast to reach the TI (which was not exposed to the stimulatory effect on bowel motility). However, failure of oral contrast to reach the TI was observed in a higher proportion of HCs (45% of HCs) than in patients (~22% of patients) so does not appear to explain the lower TI motility in patients. In patients where oral contrast reaches the colon, the oral contrast often appears to progress normally through most of the small bowel until the TI where low TI motility is observed.

It would have been useful to also measure colonic motility since it is assumed that a less motile colon partly explains the poor progress of contents from the TI to the colon. However, colonic motility occurs over a much longer time period than small bowel motility and therefore could not be captured with the current data.

A limitation of this study is that we have no report on colonic transit or colonic manometry, techniques that can be useful in confirming slow‐transit constipation and motility patterns, respectively.^42^, ^43^ However, diagnosis and management of IBS and functional bloating does not require a colonic transit study and colonic manometry is limited mainly to the research arena. Furthermore, a patient with MRI features of intestinal pseudo‐obstruction, that is, increased small bowel diameter, was excluded from the study. None of the patients included in the analysis had abnormal small bowel diameter suggestive of gross enteric dysmotility.

We also found increased right colonic diameter in patients presenting with FABD, which also may contribute to abdominal distension. Previous studies suggest that accumulation of fermentable residues determines total gas production and may in turn increase bloating and distension symptoms.^12^, ^44^ There were a higher proportion of females in the patient cohort compared with the HC cohort. It has been suggested that females have longer ascending colons than males^45^ and a reduction in intestinal tone has been found in a largely female IBS cohort.^46^ The “fecalization” pattern being studied is, however, considered to be an observation seen specifically in IBS‐C and functional bloating patients, rather than a sex‐specific occurrence (see Table S3).

Abdominal distension etiology is multifactorial. Slow colonic transit, increased luminal content of gas resulting from the fermentation of food residues and altered intestinal gas transit,^9^ consumption of more flatulent food, and/or altered microbiota have been proposed. In the last decade, however, impaired somato‐visceral reflexes causing a dyssynergia of the abdominal walls have been proposed as the main mechanism beyond severe bloating and distension in patients with functional gut disorders.^47^, ^48^ We did not specifically investigate abdominal wall movement in this study. In healthy controls, it has been shown that the muscular activity of the abdominal wall increases in response to the presence of colonic gas loads and gastric liquid loads containing nutrients to induce gastric relaxation, facilitated by viscero‐somatic reflexes.^41^, ^49^ Conversely, bloated patients (IBS‐C and FABD) demonstrated impaired abdominal contraction in response to colonic gas loads.^41^

Our study does have limitations. The sample size was small, but the study was exploratory in nature. However, we were stringent in our patient selection. We only included patients with severe functional distension and bloating. We only identify this presentation in patients fulfilling the criteria for IBS‐C or FABD; future studies should include IBS‐D and IBS‐M patients to confirm the findings are not limited to the IBS‐C subgroup. Although there was also no significant difference found between IBS‐C and FABD patients, it would be interesting to explore any possible difference between IBS‐C and FABD patients further with a larger, prospective cohort (Figure S8). Patients and healthy controls were scanned on multiple MRI platforms^50^ (see Figure S10 and Table S4). However, the impact of scanner variation may be reduced because we used intra‐patient ratios for texture analysis rather than standalone values, the image intensity was scaled within each patient, the reconstructed pixel sizes were the same on both scanners with all images of a standard used clinically for diagnosis, and the same GLCM size of 32 × 32 was used for all analyses; furthermore, motility quantification is known to be robust across various MRI platforms. Another limitation was that our study is retrospective and therefore could not exclude patients using motility influencing medication, which could potentially affect the results.

A strength of the study is the use of standard enteric MRI protocols, widely used in clinical practice, with the potential for larger future studies considering the substantial amount of clinical data available.

In summary, in this exploratory study we have demonstrated differences in terminal ileal content texture, motility metrics and right‐sided colonic diameter in patients with IBS and functional bloating presenting with severe bloating and distension. Our study may provide mechanistic insights into patients’ symptoms, supporting altered gut motility as a possible contributing factor and potentially identifying a biological marker that might influence therapeutic management.

## DISCLOSURE

Alex Menys is the founder and CEO of Motilent Ltd., a medical imaging analysis company. Stuart Taylor is a research consultant for Robarts Clinical Trials on MRI in Crohn’s disease and has shared options in Motilent. Stuart Taylor is also an NIHR senior investigator. Ruaridh Gollifer, Natalia Zarate‐Lopez, Dave Chatoor, Anton Emmanuel, and David Atkinson have no competing interests.

## AUTHOR CONTRIBUTIONS

Natalia Zarate‐Lopez, Dave Chatoor, and Anton Emmanuel collected the data. Ruaridh Gollifer and David Atkinson performed data and statistical analysis. All authors planned the study, interpreted the data, drafted the manuscript, and approved the final version of the manuscript to be submitted.

## Supporting information

Fig S1

Fig S2

Fig S3

Fig S4

Fig S5

Fig S6

Fig S7

Fig S8

Fig S9

Fig S10

Table S1

Table S2

Table S3

Table S4
