# Supplementary figures and images for: Magnetic resonance imaging assessed enteric motility and luminal content analysis in patients with severe bloating and visible distension
Source: Neurogastroenterol Motil. 2022 Apr 19;34(10):e14381. doi: 10.1111/nmo.14381 (PMC9786248; doi:10.1111/nmo.14381)

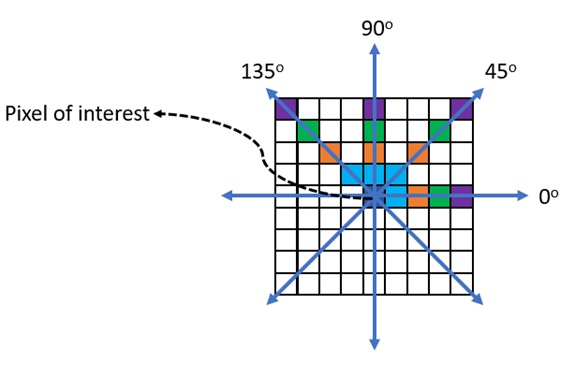

Supplement: Supplementary file 1 — Fig S1 [file NMO-34-e14381-s014.tif]

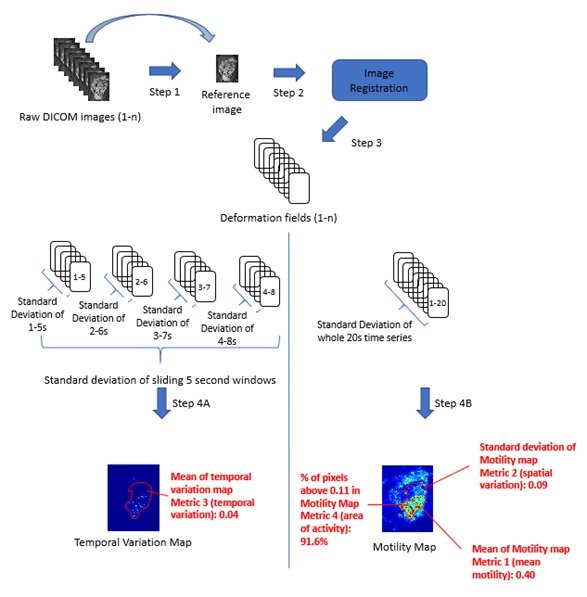

Supplement: Supplementary file 2 — Fig S2 [file NMO-34-e14381-s003.tif]

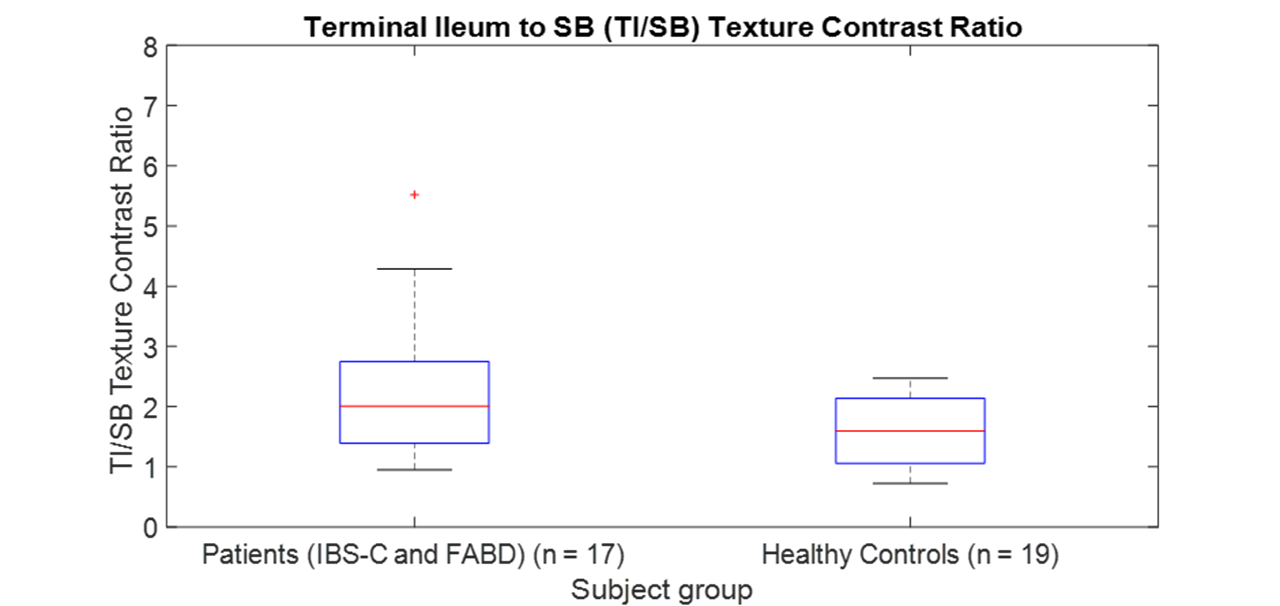

Supplement: Supplementary file 3 — Fig S3 [file NMO-34-e14381-s008.tif]

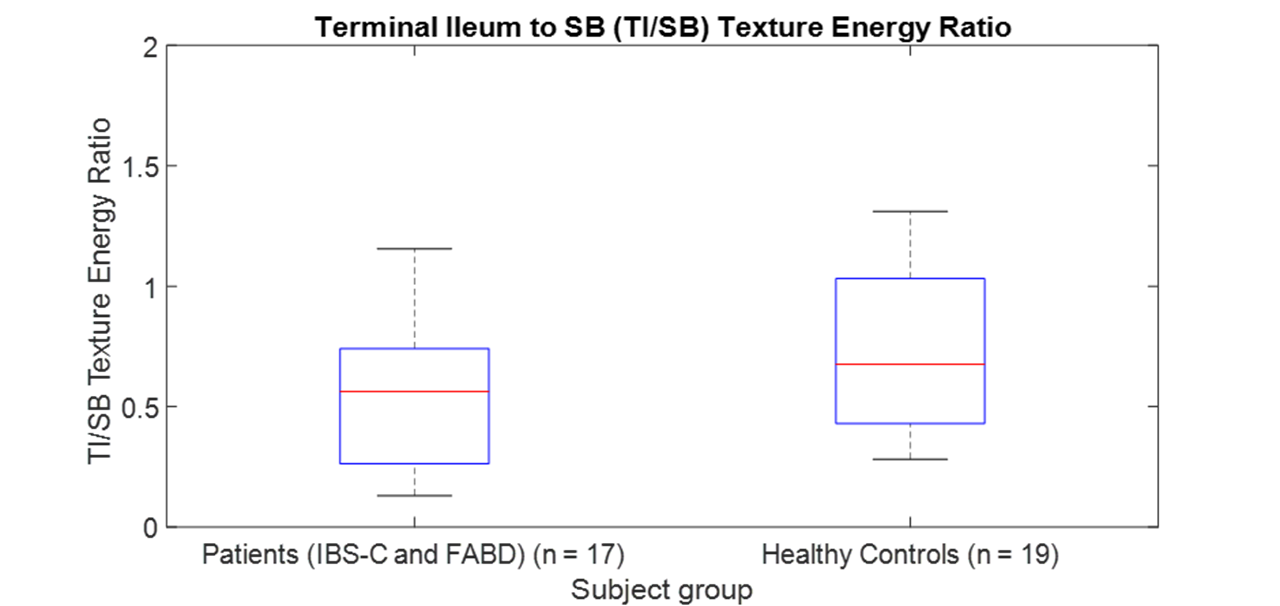

Supplement: Supplementary file 4 — Fig S4 [file NMO-34-e14381-s004.tif]

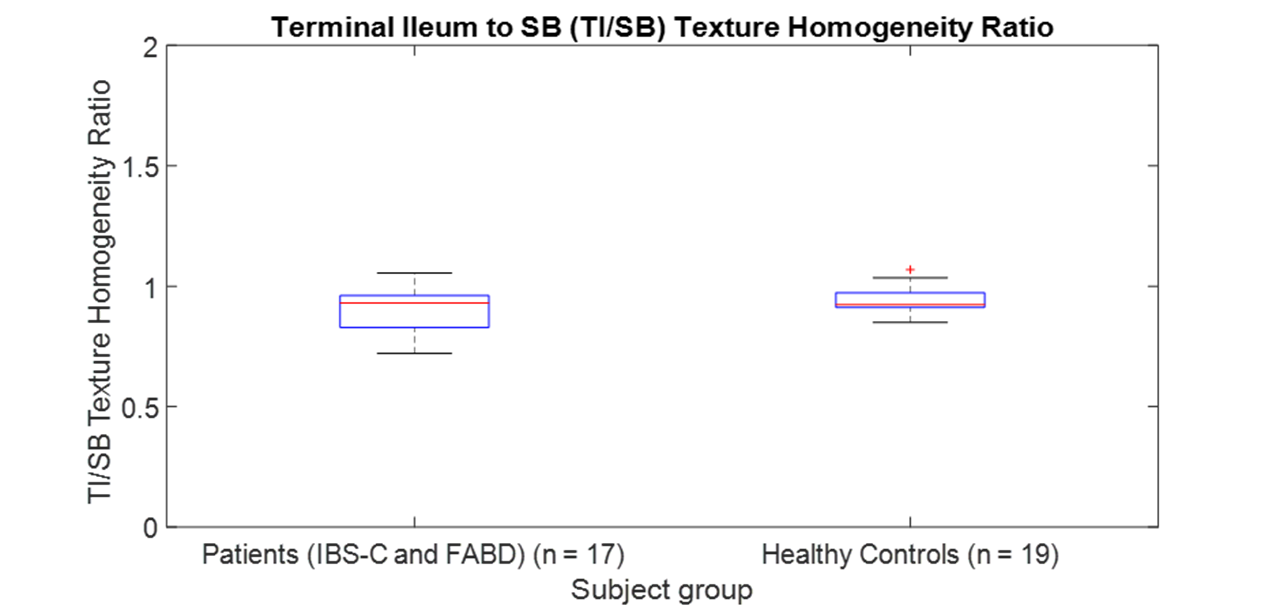

Supplement: Supplementary file 5 — Fig S5 [file NMO-34-e14381-s009.tif]

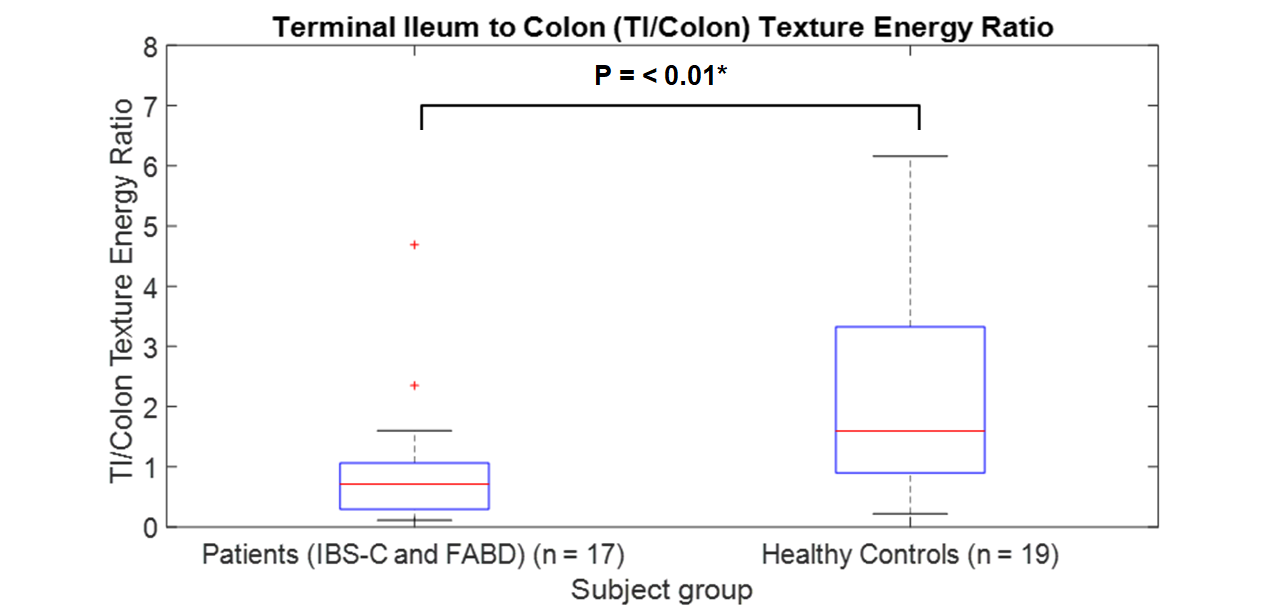

Supplement: Supplementary file 6 — Fig S6 [file NMO-34-e14381-s012.tif]

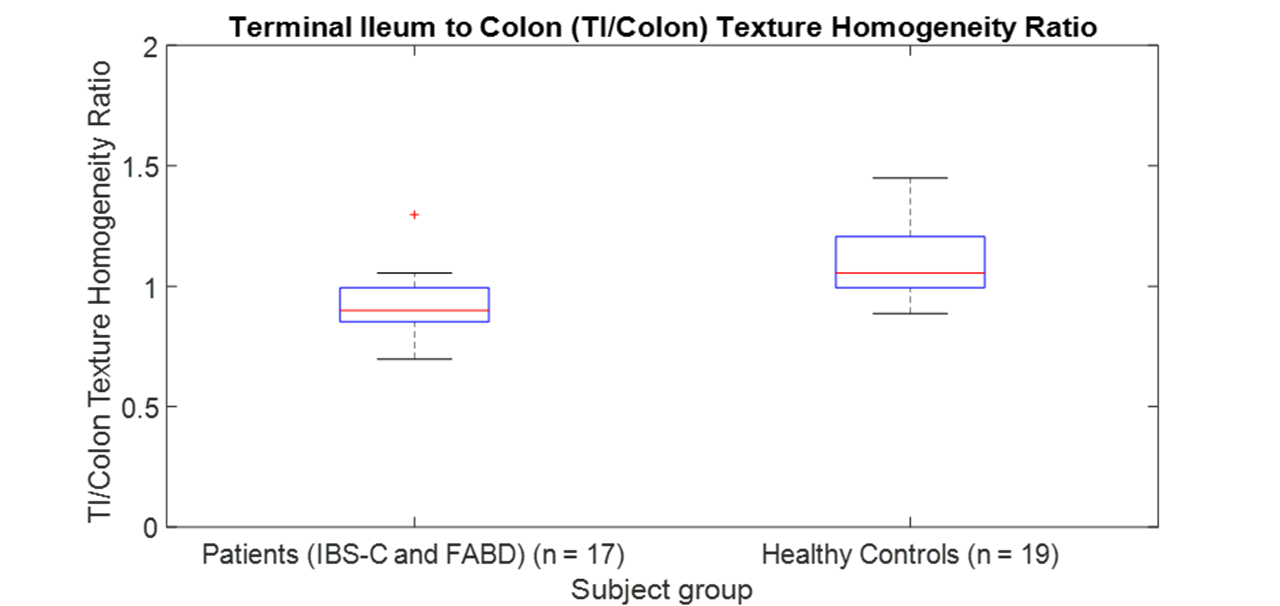

Supplement: Supplementary file 7 — Fig S7 [file NMO-34-e14381-s011.tif]

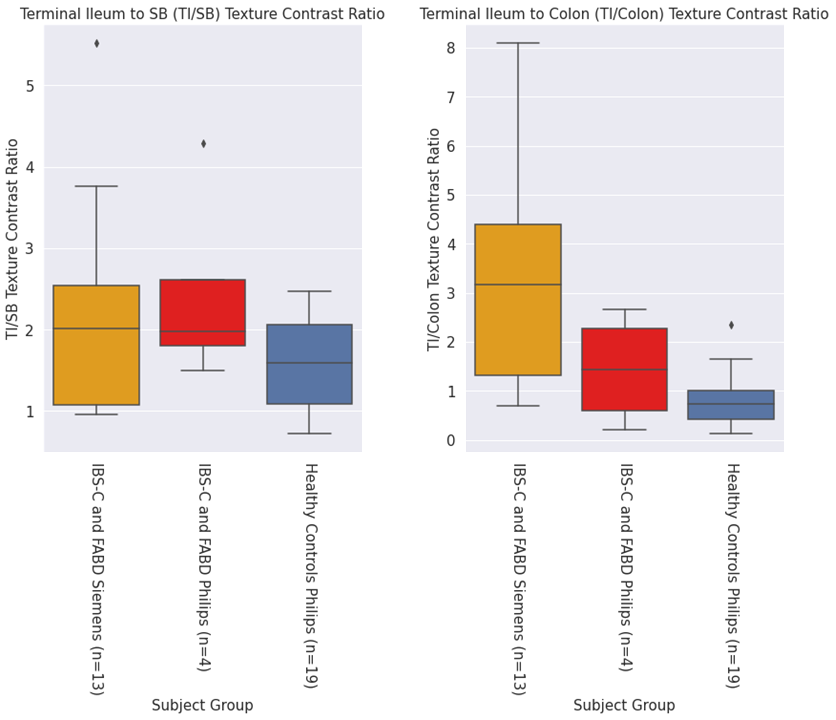

Supplement: Supplementary file 8 — Fig S8 [file NMO-34-e14381-s010.tif]

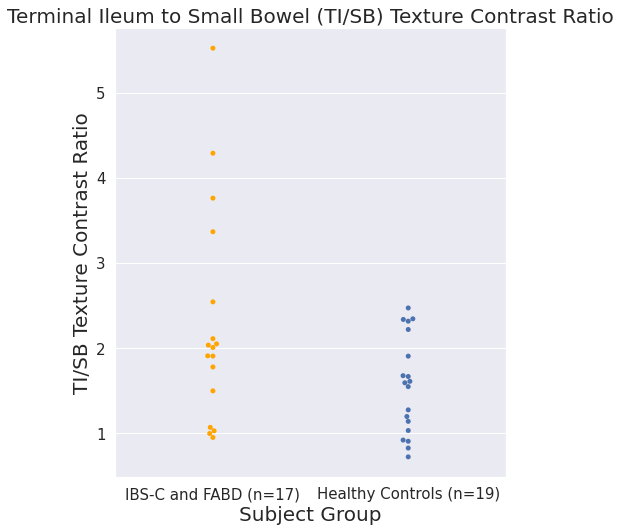

Supplement: Supplementary file 9 — Fig S9 [file NMO-34-e14381-s001.tif]

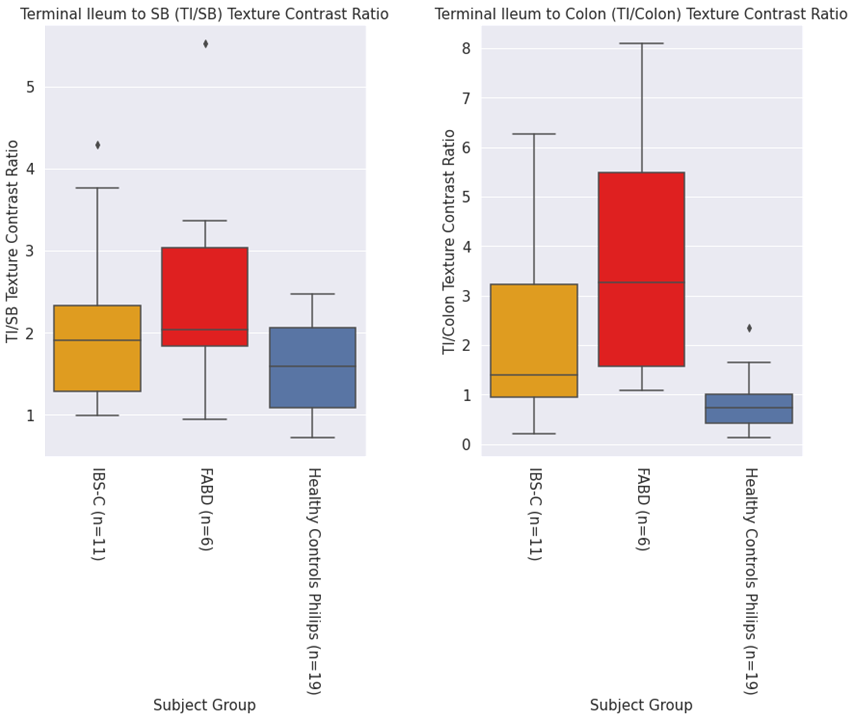

Supplement: Supplementary file 10 — Fig S10 [file NMO-34-e14381-s013.tif]
