# Supplementary material for: Magnetic resonance imaging assessed enteric motility and luminal content analysis in patients with severe bloating and visible distension
Source: Neurogastroenterol Motil. 2022 Apr 19;34(10):e14381. doi: 10.1111/nmo.14381 (PMC9786248; doi:10.1111/nmo.14381)
Supplement: Supplementary file 11 — Table S1 [file NMO-34-e14381-s006.docx]

| **Patients (n = 17)** | | | | **Healthy Controls (n = 19)** | | | **Patients vs HCs** |
| --- | --- | --- | --- | --- | --- | --- | --- |
| **Texture Analysis Summary Measures** | Median | Range | | Median | Range | | **P-values** |
|  |  | Min. | Max. |  | Min. | Max. |  |
| **TA Contrast Pixel Distances** | **Terminal Ileum to Small Bowel Ratio** | | | | | | |
| **1** | 2.01 | 0.95 | 5.52 | 1.59 | 0.72 | 2.47 | 0.08 |
| **2** | 2.34 | 0.94 | 7.28 | 1.87 | 0.54 | 4.60 | 0.08 |
| **3** | 2.56 | 0.86 | 8.52 | 1.80 | 0.51 | 6.13 | 0.15 |
| **4** | 2.72 | 0.85 | 9.22 | 1.77 | 0.51 | 8.91 | 0.16 |
|  | **Terminal Ileum to Colon Ratio** | | | | | | |
| **1** | 2.13 | 0.20 | 8.09 | 0.74 | 0.13 | 2.34 | 0.001* |
| **2** | 3.15 | 0.15 | 12.91 | 0.63 | 0.08 | 3.47 | 0.001* |
| **3** | 2.75 | 0.12 | 17.63 | 0.64 | 0.07 | 5.45 | 0.002* |
| **4** | 2.15 | 0.12 | 22.0 | 0.62 | 0.05 | 7.68 | 0.005* |
| **TA Energy Pixel Distances** | **Terminal Ileum to Small Bowel Ratio** | | | | | | |
| **1** | **0.56** | 0.13 | 1.16 | 0.68 | 0.28 | 1.31 | 0.17 |
| **2** | 0.54 | 0.12 | 1.39 | 0.69 | 0.21 | 1.45 | 0.19 |
| **3** | **0.54** | 0.14 | 1.40 | 0.66 | 0.19 | 1.44 | 0.24 |
| **4** | **0.52** | 0.15 | 1.34 | 0.62 | 0.19 | 1.86 | 0.17 |
|  | **Terminal Ileum to Colon Ratio** | | | | | | |
| **1** | 0.71 | 0.11 | 4.69 | 1.59 | 0.22 | 11.82 | 0.01* |
| **2** | **0.81** | 0.10 | 5.31 | 1.56 | 0.18 | 14.39 | 0.02 |
| **3** | **0.74** | 0.10 | 5.73 | 1.64 | 0.17 | 13.75 | 0.04 |
| **4** | **0.73** | 0.10 | 5.72 | 1.56 | 0.17 | 11.44 | 0.04 |
| **TA Homogeneity Pixel Distances** | **Terminal Ileum to Small Bowel Ratio** | | | | | | |
| **1** | **0.93** | 0.72 | 1.05 | 0.92 | 0.85 | 1.07 | 0.18 |
| **2** | **0.90** | 0.63 | 1.04 | 0.90 | 0.74 | 1.14 | 0.15 |
| **3** | **0.88** | 0.60 | 1.05 | 0.90 | 0.68 | 1.17 | 0.22 |
| **4** | **0.87** | 0.56 | 1.04 | 0.87 | 0.64 | 1.28 | 0.21 |
|  | **Terminal Ileum to Colon Ratio** | | | | | | |
| **1** | **0.90** | 0.70 | 1.30 | 1.05 | 0.89 | 1.45 | 0.001* |
| **2** | **0.89** | 0.60 | 1.44 | 1.05 | 0.78 | 1.75 | 0.006* |
| **3** | **0.88** | 0.55 | 1.57 | 1.08 | 0.72 | 1.85 | 0.02 |
| **4** | **0.81** | 0.52 | 1.63 | 1.08 | 0.66 | 1.94 | 0.03 |
