# Supplementary material for: Magnetic resonance imaging assessed enteric motility and luminal content analysis in patients with severe bloating and visible distension
Source: Neurogastroenterol Motil. 2022 Apr 19;34(10):e14381. doi: 10.1111/nmo.14381 (PMC9786248; doi:10.1111/nmo.14381)
Supplement: Supplementary file 12 — Table S2 [file NMO-34-e14381-s002.docx]

| **Texture Analysis Contrast Summary Measure** | | | |
| --- | --- | --- | --- |
| **Patients TI** | **Patients Colon** | **HCs TI** | **HCs Colon** |
| 0.48 | 2.36 | 1.34 | 2.33 |
| 0.87 | 0.63 | 0.59 | 0.74 |
| 0.31 | 0.22 | 0.42 | 0.18 |
| 1.78 | 2.44 | 0.59 | 0.91 |
| 1.31 | 0.21 | 0.31 | 0.29 |
| 1.16 | 0.54 | 0.51 | 0.68 |
| 0.57 | 0.52 | 0.50 | 0.53 |
| 1.04 | 0.24 | 0.53 | 0.60 |
| 0.79 | 0.24 | 0.35 | 0.47 |
| 0.20 | 0.29 | 0.78 | 0.51 |
| 0.59 | 0.45 | 0.42 | 2.03 |
| 0.82 | 0.26 | 0.28 | 2.11 |
| 1.03 | 0.28 | 0.25 | 1.08 |
| 1.52 | 0.19 | 0.33 | 1.24 |
| 0.25 | 0.21 | 0.36 | 0.51 |
| 1.12 | 0.19 | 0.38 | 0.23 |
| 1.09 | 0.41 | 1.02 | 0.89 |
|  |  | 0.75 | 1.11 |
|  |  | 0.45 | 2.04 |
