# Supplementary material for: Magnetic resonance imaging assessed enteric motility and luminal content analysis in patients with severe bloating and visible distension
Source: Neurogastroenterol Motil. 2022 Apr 19;34(10):e14381. doi: 10.1111/nmo.14381 (PMC9786248; doi:10.1111/nmo.14381)
Supplement: Supplementary file 13 — Table S3 [file NMO-34-e14381-s005.docx]

| **Patient Females (n = 16)** | | | | **Healthy Control Females (n = 7)** | | | **Patients vs HCs** |
| --- | --- | --- | --- | --- | --- | --- | --- |
| **Texture Analysis Summary Measures** | Median | Range | | Median | Range | | **P-values** |
|  |  | Min. | Max. |  | Min. | Max. |  |
| **TA Contrast Pixel Distances** | **Terminal Ileum to Small Bowel Ratio** | | | | | | |
| **1** | 2.02 | 0.95 | 5.52 | 1.20 | 0.83 | 2.22 | 0.05 |
| **2** | 2.37 | 0.94 | 7.28 | 1.58 | 0.80 | 3.14 | 0.10 |
| **3** | 2.81 | 0.86 | 8.52 | 1.59 | 0.92 | 3.8 | 0.15 |
| **4** | 3.18 | 0.85 | 9.22 | 1.56 | 0.83 | 5.07 | 0.19 |
|  | **Terminal Ileum to Colon Ratio** | | | | | | |
| **1** | 2.39 | 0.69 | 8.09 | 0.7 | 0.23 | 2.34 | **0.01*** |
| **2** | 3.46 | 0.6 | 12.91 | 0.6 | 0.13 | 3.47 | **0.01*** |
| **3** | 3.76 | 0.51 | 17.63 | 0.67 | 0.09 | 5.45 | 0.02 |
| **4** | 3.64 | 0.42 | 22 | 0.73 | 0.09 | 7.68 | 0.04 |
| **TA Energy Pixel Distances** | **Terminal Ileum to Small Bowel Ratio** | | | | | | |
| **1** | 0.5 | 0.13 | 1.16 | 0.78 | 0.36 | 1.09 | 0.23 |
| **2** | 0.48 | 0.12 | 1.39 | 0.82 | 0.32 | 1.04 | 0.28 |
| **3** | 0.49 | 0.14 | 1.4 | 0.75 | 0.32 | 1.07 | 0.33 |
| **4** | 0.47 | 0.15 | 1.34 | 0.62 | 0.3 | 1.86 | 0.14 |
|  | **Terminal Ileum to Colon Ratio** | | | | | | |
| **1** | 0.69 | 0.11 | 2.35 | 1.67 | 0.22 | 3.84 | 0.06 |
| **2** | 0.75 | 0.1 | 2.77 | 1.68 | 0.18 | 4.2 | 0.09 |
| **3** | 0.73 | 0.1 | 3.09 | 1.66 | 0.17 | 4.6 | 0.13 |
| **4** | 0.72 | 0.1 | 3.36 | 1.56 | 0.17 | 4.58 | 0.09 |
| **TA Homogeneity Pixel Distances** | **Terminal Ileum to Small Bowel Ratio** | | | | | | |
| **1** | 0.92 | 0.72 | 1.05 | 0.96 | 0.88 | 1.04 | 0.16 |
| **2** | 0.90 | 0.63 | 1.04 | 0.96 | 0.80 | 1.01 | 0.23 |
| **3** | 0.87 | 0.6 | 1.05 | 0.91 | 0.76 | 1.03 | 0.36 |
| **4** | 0.86 | 0.56 | 1.04 | 0.83 | 0.75 | 1.28 | 0.23 |
|  |  | | | | | | |
| **1** | 0.9 | 0.7 | 1.05 | 1.06 | 0.89 | 1.28 | **0.005*** |
| **2** | 0.88 | 0.6 | 1.14 | 1.05 | 0.78 | 1.34 | 0.02 |
| **3** | 0.87 | 0.55 | 1.23 | 1.01 | 0.72 | 1.42 | 0.06 |
| **4** | 0.8 | 0.52 | 1.3 | 1.02 | 0.69 | 1.48 | 0.06 |
