# Supplementary material for: Magnetic resonance imaging assessed enteric motility and luminal content analysis in patients with severe bloating and visible distension
Source: Neurogastroenterol Motil. 2022 Apr 19;34(10):e14381. doi: 10.1111/nmo.14381 (PMC9786248; doi:10.1111/nmo.14381)
Supplement: Supplementary file 14 — Table S4 [file NMO-34-e14381-s007.docx]

| **Patients Philips Achieva data (n = 4)** | | | | **Healthy Control Philips Achieva data (n = 19)** | | | **Patients vs HCs** |
| --- | --- | --- | --- | --- | --- | --- | --- |
| **Texture Analysis Summary Measures** | Median | Range | | Median | Range | | **P-values** |
|  |  | Min. | Max. |  | Min. | Max. |  |
| **TA Contrast Pixel Distances** | **Terminal Ileum to Small Bowel Ratio** | | | | | | |
| **1** | 1.98 | 1.50 | 4.29 | 1.59 | 0.72 | 2.47 | 0.26 |
| **2** | 2.73 | 1.74 | 6.03 | 1.87 | 0.54 | 4.60 | 0.05 |
| **3** | 3.29 | 1.80 | 8.08 | 1.80 | 0.51 | 6.13 | 0.10 |
| **4** | 3.45 | 1.83 | 9.04 | 1.77 | 0.51 | 8.91 | 0.16 |
|  | **Terminal Ileum to Colon Ratio** | | | | | | |
| **1** | 1.43 | 0.20 | 2.66 | 0.74 | 0.13 | 2.34 | 0.49 |
| **2** | 1.95 | 0.15 | 3.76 | 0.63 | 0.08 | 3.47 | 0.27 |
| **3** | 1.88 | 0.12 | 6.59 | 0.64 | 0.07 | 5.45 | 0.27 |
| **4** | 1.79 | 0.12 | 7.71 | 0.62 | 0.05 | 7.68 | 0.24 |
| **TA Energy Pixel Distances** | **Terminal Ileum to Small Bowel Ratio** | | | | | | |
| **1** | 0.69 | 0.15 | 0.80 | 0.68 | 0.28 | 1.31 | 0.47 |
| **2** | 0.67 | 0.15 | 0.73 | 0.69 | 0.21 | 1.45 | 0.49 |
| **3** | 0.67 | 0.14 | 0.74 | 0.66 | 0.19 | 1.44 | 0.54 |
| **4** | 0.69 | 0.15 | 0.78 | 0.62 | 0.19 | 1.86 | 0.84 |
|  | **Terminal Ileum to Colon Ratio** | | | | | | |
| **1** | 0.93 | 0.47 | 4.69 | 1.59 | 0.22 | 11.82 | 0.44 |
| **2** | 0.98 | 0.45 | 5.31 | 1.56 | 0.18 | 14.39 | 0.49 |
| **3** | 0.92 | 0.42 | 5.73 | 1.64 | 0.17 | 13.75 | 0.49 |
| **4** | 0.95 | 0.42 | 5.72 | 1.56 | 0.17 | 11.44 | 0.54 |
| **TA Homogeneity Pixel Distances** | **Terminal Ileum to Small Bowel Ratio** | | | | | | |
| **1** | 0.93 | 0.75 | 0.94 | 0.92 | 0.85 | 1.07 | 0.78 |
| **2** | 0.91 | 0.66 | 0.92 | 0.90 | 0.74 | 1.14 | 0.54 |
| **3** | 0.89 | 0.61 | 0.90 | 0.90 | 0.68 | 1.17 | 0.44 |
| **4** | 0.90 | 0.56 | 0.92 | 0.87 | 0.64 | 1.28 | 0.78 |
|  | **Terminal Ileum to Colon Ratio** | | | | | | |
| **1** | 0.97 | 0.88 | 1.30 | 1.05 | 0.89 | 1.45 | 0.31 |
| **2** | 0.97 | 0.85 | 1.44 | 1.05 | 0.78 | 1.75 | 0.54 |
| **3** | 0.95 | 0.79 | 1.57 | 1.08 | 0.72 | 1.85 | 0.69 |
| **4** | 0.87 | 0.80 | 1.63 | 1.08 | 0.66 | 1.94 | 0.39 |
